# Systemic lupus erythematosus associated with type 4 renal tubular acidosis: a case report and review of the literature

**DOI:** 10.1186/1752-1947-5-114

**Published:** 2011-03-24

**Authors:** Haldane Porteous, Nadia Morgan, Julio Lanfranco, Monica Garcia-Buitrago, Larry Young, Oliver Lenz

**Affiliations:** 1 Department of Internal Medicine, University of Miami/Jackson Memorial Hospital, 1611 NW 12th Avenue, Miami, FL 33136, USA; 2 Department of Internal Medicine, State University of New York Downstate Medical Center, 450 Clarkson Avenue, Brooklyn, NY 11203, USA; 3 Department of Pathology, University of Miami/Jackson Memorial Hospital, 1611 NW 12th Avenue, Miami, FL 33136, USA; 4 Department of Rheumatology, Miami Veterans Affairs Medical Center, 1201 NW 16th Street, Miami, FL 33125, USA; 5 Department of Nephrology, University of Miami/Jackson Memorial Hospital, 1611 NW 12th Avenue, Miami, FL 33136, USA

## Abstract

**Introduction:**

Type 4 renal tubular acidosis is an uncommon clinical manifestation of systemic lupus erythematosus and has been reported to portend a poor prognosis. To the best of our knowledge, this is the first case report which highlights the successful management of a patient with systemic lupus erythematosus complicated by type 4 renal tubular acidosis who did not do poorly.

**Case presentation:**

A 44-year-old Hispanic woman developed a non-anion gap hyperkalemic metabolic acidosis consistent with type 4 renal tubular acidosis while being treated in the hospital for recently diagnosed systemic lupus erythematosus with multi-organ involvement. She responded well to treatment with corticosteroids, hydroxychloroquine and mycophenolate mofetil. Normal renal function was achieved prior to discharge and remained normal at the patient's one-month follow-up examination.

**Conclusion:**

This case increases awareness of an uncommon association between systemic lupus erythematosus and type 4 renal tubular acidosis and suggests a positive impact of early diagnosis and appropriate immunosuppressive treatment on the patient's outcome.

## Introduction

Inability of the kidney either to excrete sufficient net acid or to retain sufficient bicarbonate results in a group of disorders known as renal tubular acidoses (RTAs) [1]. These are normal anion gap hyperchloremic acidoses. In the traditional classification, type 4 is the only variant associated with hyperkalemia. Compared to the other distal RTAs, in type 4 RTA, the collecting duct fails to excrete both protons and potassium. Such a scenario arises when there is a quantitative or qualitative aldosterone deficiency or a genetic or acquired molecular defect in the relevant transporters. Aldosterone activity is necessary for adequate sodium reabsorption by the epithelial sodium channels. These channels are located on the luminal surface of principal cells in the terminal portions of the nephron. Under normal conditions, they generate a lumen-negative potential, which is essential for potassium and proton secretion [2].

RTA is a rare complication of systemic lupus erythematosus (SLE) and can pose a diagnostic dilemma [3]. If inappropriately treated, chronic serum acidity ensues, predisposing to growth retardation, nephrolithiasis, bone disease, chronic kidney disease and even end-stage renal disease. Type 4 RTA is less commonly associated with SLE than type 1. Patients with type 4 RTA usually have higher SLE disease activity index (SLEDAI) scores [4].

We report a case of a patient with a high SLEDAI score and type 4 RTA secondary to SLE who received prompt and appropriate treatment and did not do poorly.

## Case presentation

A 44-year-old Hispanic woman presented to the hospital with a six-month history of generalized weakness, weight loss of 40 pounds over a four-month period and a two-week history of progressively worsening dyspnea. She had had hypertension for five years for which she was being treated with lisinopril but had been noncompliant for over three years because of financial constraints. The patient denied any alcohol or illicit drug use and was a lifelong non-smoker.

On admission, her blood pressure was 102/66 mm/Hg, her temperature was 37°C, her respiratory rate was 24 breaths/minute, her oxygen saturation level was 92% on room air and her pulse rate was 88 beats/minute. A physical examination revealed an ill-looking woman with mucosal pallor, generalized wasting and non-tender, rubbery axillary and inguinal lymphadenopathy. There was no evidence of cyanosis, digital clubbing, pitting edema, skin rash or joint deformities. Her abdomen was mildly distended but non-tender, with no organomegaly detected. Dullness to percussion and decreased breath sounds over the left base were noted on the respiratory system examination. The cardiovascular and neurological examinations were unremarkable.

Initial laboratory investigations (Table 1) revealed anemia, leukopenia, elevated blood urea nitrogen and elevated serum creatinine. Urinalysis showed trace proteinuria. Chest radiography revealed bilateral pleural effusions that were determined to be exudative in nature on the basis of thoracocentesis. There was a high index of suspicion for malignancy; however, the results of chest, abdomen and pelvis computed tomography scans and an axillary lymph node biopsy did not confirm this. Human immunodeficiency virus and tuberculin skin tests were negative.

**Table 1:** Laboratory investigations on admission to Jackson Memorial Hospital

Hemoglobin, 6.6 g/dl		Na^+^, 131 mM/l
Hematocrit, 20.9%		K^+^, 5.7 mM/l
Platelets, 544 × 10^9^/l		Cl^-^, 105 mM/l
White blood cell count, 3.5 × 10^9^/l	Neutrophils, 80.4%	HCO_3_, 18 mM/l
	Lymphocytes, 16.0%	Blood urea nitrogen, 60 mg/dl
	Monocytes, 2.1%	Creatinine, 1.69 mg/dl
	Eosinophils, 0.3%	

On day four of admission, she developed acute inflammatory arthritis of the elbows and knees. A serological workup revealed high anti-nuclear antibody, anti-double-stranded DNA antibody and anti-Smith antibody titers, with low complement 3 levels. An active sediment was noted on the basis of urinalysis (Table 2). The patient's renal impairment persisted, and her glomerular filtration rate was estimated to be 53 ml/min/1.73 m^2^. A review of blood results since admission showed evidence of documented leukopenia on more than two occasions.

**Table 2:** Urinalysis results from day 4 of admission

pH 5	White blood cell count, 16 per high power field
24-hour urinary protein, 0.81 g/day	Hyaline cast, zero to two per high-power field
Red blood cell count, 27 per high-power field	Squamous epithelial cells, one per high-power field

On day 5 of admission, the patient experienced dyspnea and pleuritic chest pain. Reaccumulated pleural effusions were noted on a chest radiograph, and an echocardiogram showed evidence of pericardial effusion. Arterial blood gas interpreted in conjunction with a corresponding basal metabolic panel (Table 3) revealed the presence of a non-anion gap hyperkalemic metabolic acidosis consistent with type 4 RTA. Analysis of morning serum sample showed a serum aldosterone level less than 1 ng/dl.

**Table 3:** Arterial blood gas and basic metabolic panel results from day 5 of admission

pH 7.34	Na^+^, 145 mM/l
HCO_3_, 14 mM/l	K^+^, 5.5 mM/l
CO_2_, 26 mmHg	Cl^-^, 120 mM/l
pO_2_, 96 mmHg	HCO_3_, 19 mM/l
	Blood urea nitrogen, 67 mg/dl
	Creatinine, 1.09 mg/dl

**Figure 1:**
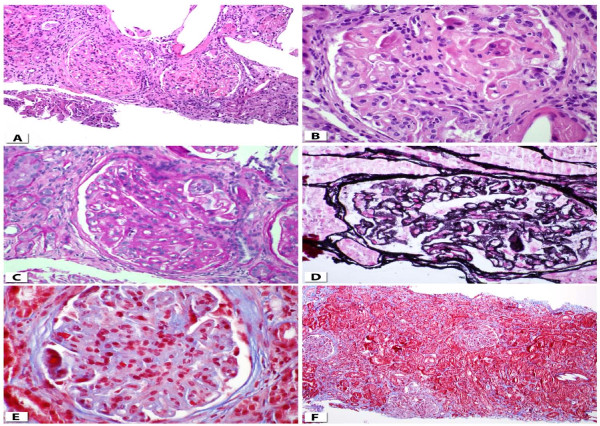
**Photomicrographs of the renal biopsy**. **(a)** Lupus nephritis class IV-G(A) + V (hematoxylin and eosin stain; original magnification, × 200). **(b) **Glomerulus showing global endocapillary proliferation, fibrinoid necrosis, neutrophilic infiltration and capillary wall thickening (hematoxylin and eosin stain; original magnification, × 600). **(c) **Glomerulus showing mesangial expansion with hypercellularity and occlusion of peripheral lumina (periodic acid-Schiff stain; original magnification, × 600). **(d) **Glomerulus showing thickened basement membranes with numerous spikes and occasional dual contour (methenamine silver stain; original magnification, × 600). **(e) **Glomerulus showing areas of bright red fibrinoid necrosis (trichrome stain; original magnification, × 600). **(f) **Minimal tubulointerstitial fibrosis (trichrome stain; original magnification, × 100).

The renal ultrasound obtained showed normal kidney morphology and no evidence of nephrolithiasis. A renal biopsy was done, which revealed diffuse global proliferative and membranous glomerulonephritis. This was consistent with lupus nephritis Renal Pathology Society/International Society of Nephrology 2003 class IV-G(A) and V, moderate activity index 9/24, minimal chronicity index 1/12; minimal tubulointerstitial fibrosis and acute tubular necrosis (Figure 1).

She was diagnosed with SLE complicated by a generalized lupus flare, with a SLEDAI score of 29. Overall during this single admission, she demonstrated six of the 11 American College of Rheumatology criteria used in the diagnosis of SLE.

**Figure 2:**
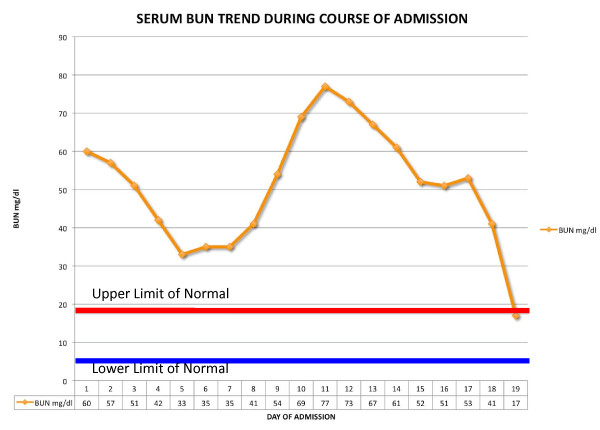
**Graph showing trend of serum urea during admission**.

**Figure 3:**
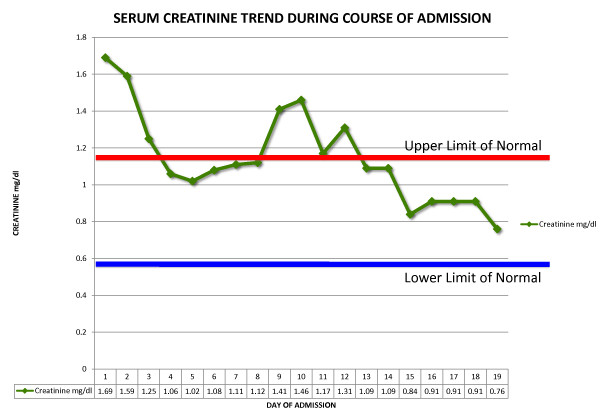
**Graph showing trend of serum creatinine during admission**.

**Figure 4:**
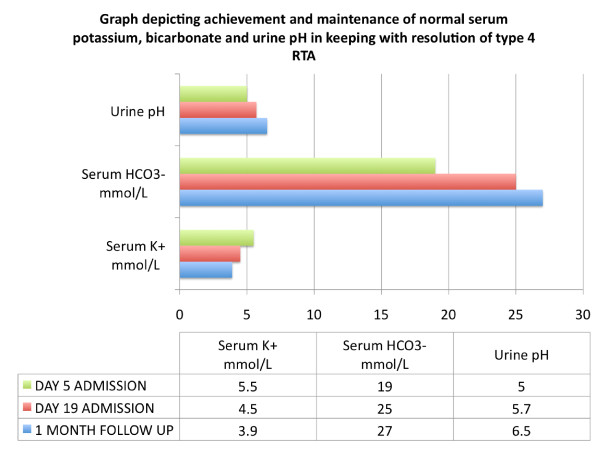
**Graph depicting achievement and maintenance of normal serum potassium, bicarbonate and urine pH in keeping with resolution of type 4 renal tubular acidosis**.

The patient was treated with a course of hydroxychloroquine and intravenous methylprednisone 1 g daily for three days. Thereafter she was placed on a prednisone, mycophenolate mofetil and hydroxychloroquine regimen. Complete resolution of the renal impairment (Figures 2 and 3) and type 4 RTA (Figure 4) was achieved. She was discharged after 19 days to follow-up in the Rheumatology and Nephrology clinics. Her renal function remained normal at the one-month follow-up clinic visit.

## Discussion

The clinical manifestations of SLE are many and varied, making it a plausible component of many differential diagnoses [5]. It is one of several diseases known as "the great imitators" because it often mimics other diseases. RTA is a medical condition that involves an accumulation of acid in the body due to a failure of the kidneys to appropriately acidify the urine [1].

As many as 60% of adults with SLE develop overt renal abnormalities, and 10% to 15% of patients with lupus nephritis progress to end-stage renal failure [6]. RTA is rarely associated with SLE and, if present, is more commonly of the type 1 than the type 4 variety [4]. It likely represents the consequence of significant tubulointerstitial damage, which should signal the need for rapid treatment of the underlying lupus nephritis to avoid future renal insufficiency [7].

In the setting of a hyperkalemic normal anion gap metabolic acidosis and a urine pH less than 5.5, the clinician should have a high index of suspicion for the presence of type 4 RTA. Conversely, a diagnosis of incomplete type 1 RTA may be entertained in patients with hyperkalemia and a urine pH which is persistently greater than 5.5 [8].

The presence of type 4 RTA can be confirmed by calculating the transtubular potassium concentration gradient (TTKG) [9]. TTKG is an index of the potassium-secreting activity of the cells in the distal tubule. In normal individuals, hyperkalemia is associated with increased aldosterone secretion and distal potassium excretion, leading to a high TTKG level (usually greater than 10). A TTKG level less than 7 is highly suggestive of hyporeninemic hypoaldosteronism probably due to tubulointerstitial damage [10]. Our patient's calculated TTKG was 5.3 (Table 4).

**Table 4:** Calculation of transtubular gradient

Urine sodium, 59 mM/l	Transtubular gradient
Urine potassium, 38 mM/l	Urine K/plasma K
Urine osmolality, 405 mOsm/kg	Urine osmolality/plasma osmolality
Plasma potassium, 5.1 mM/l	
Plasma osmolality, 288 mOsm/kg	Patient's transtubular gradient = 5.3

Other causes of type 4 RTA were ruled out. Our patient had a normal cortisol level of 14.4 μg/dl, making adrenal insufficiency unlikely. She divulged no history of taking non-steroidal anti-inflammatory drugs, potassium-sparing diuretics, heparin, trimethoprim or angiotensin receptor blocker use. A history of angiotensin-converting enzyme inhibitor use was elicited; however, she was completely non-compliant with the same for the past three years, making this an unlikely cause.

Li *et al*. [4] noted the association between type 4 RTA and SLE in patients with high SLEDAI scores. They found that the degree of hyperkalemia was correlated with a high SLEDAI score and that these patients had a poor outcome of chronic renal insufficiency requiring hemodialysis at admission or resulting in death. Patients with type 4 RTA have more extensive tubular damage stemming from aggressive nephritis associated with more aggressive systemic manifestations of SLE. Thus, the presence of type 4 RTA is an indicator of more aggressive SLE. In our patient, hyperkalemia and a high SLEDAI score of 29 were noted; however, she did not do poorly. Her type 4 RTA and significant renal impairment resolved completely after two weeks of appropriate therapy.

## Conclusion

To the best of the authors' knowledge this is the first reported case of a patient with SLE who had a high SLEDAI score and type 4 RTA secondary to lupus nephritis, yet did not do poorly. This case emphasizes the fact that early diagnosis and appropriate treatment can indeed result in the desired positive outcome.

## Abbreviations

RTA: renal tubular acidosis; SLE: systemic lupus erythematosus; SLEDAI: systemic lupus erythematosus disease activity index; TTKG: transtubular potassium gradient.

## Consent

Written informed consent was obtained from our patient for publication of this case

report and any accompanying images. A copy of the written consent is available for review by the Editor-in-Chief of this journal.

## Competing interests

The authors declare that they have no competing interests.

## Authors' contributions

HP and NM collected the data and drafted the manuscript. HP and JL contributed to the treatment of the patient. LY and OL participated in critical revision of the report and helped draft the manuscript. All authors read and approved the final manuscript.
